# Benefits of umbilical cord milking versus delayed cord clamping on neonatal outcomes in preterm infants: A systematic review and meta-analysis

**DOI:** 10.1371/journal.pone.0201528

**Published:** 2018-08-30

**Authors:** Nobuhiko Nagano, Makoto Saito, Takahiro Sugiura, Fumiko Miyahara, Fumihiko Namba, Erika Ota

**Affiliations:** 1 Department of Pediatrics and Child Health, Nihon University School of Medicine, Itabashi, Tokyo, Japan; 2 Department of Pediatrics, University of Tsukuba, Tsukuba, Ibaraki, Japan; 3 Department of Pediatrics, Toyohashi Municipal Hospital, Toyohashi, Aichi, Japan; 4 Division of Pediatrics and Perinatology, Tottori University Faculty of Medicine, Yonago, Tottori, Japan; 5 Department of Pediatrics, Saitama Medical Center, Saitama Medical University, Kawagoe, Saitama Japan; 6 Global Health Nursing, Graduate School of Nursing Science, St. Luke’s International University, Chuo, Tokyo, Japan; RCSI & UCD Malaysia Campus (formerly Penang Medical College), MALAYSIA

## Abstract

**Background:**

Optimum timing of umbilical cord clamping has not been established in preterm infants.

**Objectives:**

We compared the short- and long-term effects of umbilical cord milking (UCM) versus delayed cord clamping (DCC) on infants born at less than 37 weeks of gestation.

**Search methods:**

A systematic review and meta-analysis was conducted according to the “Preferred Reporting Items for Systematic Reviews and Meta-Analyses” statement. We searched CINAHL, CENTRAL, EMBASE, MEDLINE, PubMed and ClinicalTrials.gov for relevant randomized controlled trials (RCTs).

**Selection criteria:**

We included individual and clustered RCTs comparing UCM to DCC for infants born before 37 weeks of gestation.

**Data collection and analysis:**

Four reviewers independently assessed trial quality and eligibility for inclusion.

**Main results:**

Two trials (255 preterm infants, 23 0/7 to 32 6/7 weeks of gestation) were included in the analysis. UCM was associated with fewer intraventricular hemorrhages (IVHs) (two trials, 255 infants; relative risk [RR] 0.45, 95% confidence interval [CI] 0.20 to 0.98, low quality of evidence) and UCM was an increased proportion of infants with a Bayley score at 2 years of age (two trials, 174 infants; Cognitive: RR 1.14, 95% CI 1.03 to 1.26, Language: RR 1.24, 95% CI 1.03 to 1.49, low quality of evidence) compared to DCC.

**Conclusions:**

UCM wasn’t reduced in-hospital mortality and need for transfusion compared to DCC. But our study suggests that UCM may lower the risk of IVH and improve certain neurodevelopmental outcomes compared to DCC in preterm infants.

## Introduction

The World Health Organization recommends delayed cord clamping (DCC), defined as a delay of 30 seconds or longer after birth, as standard delivery room care for infants who do not need resuscitation [1]. Indeed, a delay in cord clamp between 30 and 120 seconds in preterm infants born at less than 37 weeks of gestation reduced the risk of intraventricular hemorrhage (IVH) (all grades) and the need for blood transfusion, provided better circulatory stability, and lowered the risk for developing necrotizing enterocolitis compared to immediate cord clamping (ICC) [2]. Moreover, a recent systematic review and meta-analysis of DCC versus ICC for preterm infants concluded that DCC reduces hospital mortality [3]. However, most preterm neonates require resuscitation immediately after birth, and DCC may impede resuscitation, so the optimum timing of umbilical cord clamping has not been established. An alternative to DCC is umbilical cord milking (UCM), in which the unclamped umbilical cord is squeezed and blood is massaged toward the preterm infant with the intention of transfusing blood. This is repeated up to four times before the cord is clamped. For infants born at less than 29 weeks of gestation, UCM reduces the need for red blood cell transfusion compared to ICC [4]. Furthermore, UCM can be performed expeditiously within current resuscitation timeline recommendations before clamping the umbilical cord, and thus may be more beneficial than DCC [5].

The International Liaison Committee of Resuscitation guideline states that DCC for longer than 30 seconds is valuable for both term and preterm infants who do not require immediate resuscitation at birth. However, the guideline also noted a lack of information regarding the safety of rapid changes in blood volume for extremely preterm infants, and did not recommend the routine use of UCM for infants born at less than 29 weeks of gestation outside of a medical facility [6]. Therefore, we compared the effects of DCC versus UCM in preterm infants on in-hospital mortality, need for transfusion, initial hematocrit level, hemoglobin (Hb) level at birth, maximum serum bilirubin, IVH risk, developmental quotient at 2 and 3.5 years of age, polycythemia, mean blood pressure at 4 hours, urine output over the first 24 hours, duration of phototherapy, necrotizing enterocolitis, oxygen dependency at 36 weeks, sepsis, length of hospital stay, spontaneous intestinal perforation, need for pressors, and Apgar score at 5 min.

## Methods

We performed a systematic review of randomized controlled trials (RCTs) according to the *Cochrane Handbook for Systematic Reviews of Interventions* [7]. The protocol was registered on PROSPERO, an international prospective registry of systematic reviews (registration number: CRD42016049756). We adhered to the reporting guidelines described in the “Preferred Reporting Items for Systematic Reviews and Meta-Analyses (PRISMA)” statement [8].

### Inclusion criteria

All RCTs comparing UCM (intervention) to DCC were considered. Keywords were “umbilical cord milking” (or UCM), “delayed cord clamping” (or DCC), “preterm neonate,” “blood transfusion,” “jaundice,” and “intraventricular hemorrhage” (or IVH).

### Search strategy

We searched the CINAHL, CENTRAL, EMBASE, MEDLINE via Ovid SP, PubMed, and ClinicalTrials.gov databases on March 14, 2018 with no date/time, language, document type, and publication status limitations. Search terms were gathered from expert opinion, literature reviews, and controlled vocabulary (Medical Subject Headings, Excerpta Medica Tree, and CINAHL headings).

### Identification of studies

An information specialist conducted the searches, and the reviewers conducted supplemental searches manually. No language restrictions were applied, and in the case of unpublished or ongoing trials, the authors of those studies were contacted for further information. Four of the authors (NN, MS, TS, and FM) independently assessed all studies identified in the search for further review. Disagreements were resolved by discussion between the four authors or by consulting with a third assessor (FN).

**Fig 1:**
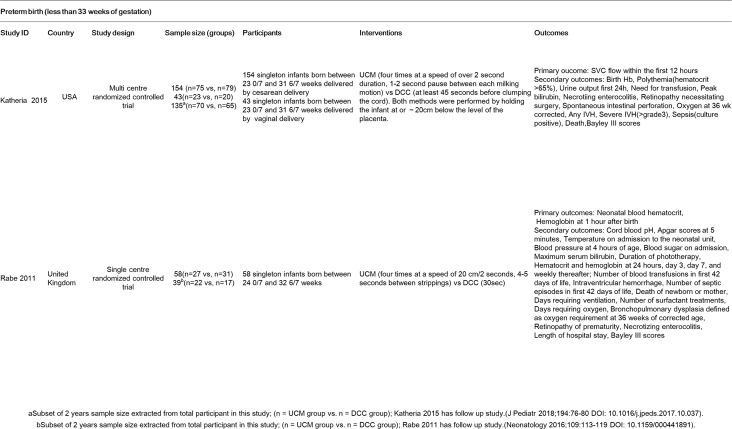
Characteristic of included studies (randomized controlled trials).

Four of the reviewers used piloted data extraction forms to collect basic study information and details on participants, control interventions, treatments, and outcomes (Fig 1).

### Data analysis

Data were analyzed using Review Manager software (RevMan 2014 http://community.cochrane.org/tools/review-production-tools/revman-5). We defined in-hospital mortality, need for transfusion, and initial hematocrit level as primary outcomes. We also defined 15 secondary outcomes: Hb level at birth, maximum serum bilirubin, any IVH, developmental quotient at 2 and 3.5 years of age, polycythemia, mean blood pressure at 4 hours, urine output in the first 24 hours, duration of phototherapy, necrotizing enterocolitis, oxygen dependence at 36 weeks, sepsis, length of hospital stay, spontaneous intestinal perforation, need for pressors, and Apgar score at 5 min. Differences in dichotomous data are expressed as risk ratios (RRs) while differences in continuous data are expressed as weighted mean difference or standardized mean difference (MD). We performed random-effects meta-analysis, and the results are presented as the average intervention effect with 95% confidence intervals (CIs). Statistical heterogeneity was estimated by the I^2^ test. A value of I^2^ >75% is considered substantial heterogeneity and 30% to 60% may represent moderate heterogeneity. If there were only a few studies or if the sample size was small, we used a fixed-effect model, since random-effects models provide poor estimates of the intervention effects distribution.

### Assessment of bias risk in included studies

Four review authors (NN, MS, TS, and FM) independently assessed the risk of bias for each included study using the criteria outlined in the *Cochrane Handbook for Systematic Reviews of Interventions* [7]. Any disagreement was resolved by discussion between the reviewers or by consulting with a third assessor (FN).

### Quality of evidence (GRADE)

To rate the quality of evidence, we used the GRADEpro Guideline Development Tool (https://gradepro.org) summary of findings template. Studies were assessed according to the Grades of Recommendation, Assessment, Development, and Evaluation (GRADE) Working Group, and quality of evidence was assessed according to the GRADE’s five downgraded criteria: risk of bias, inconsistency, indirectness, imprecision, and publication bias [9]. A quality rating was given for the following outcomes: neonatal intensive care unit mortality, initial hematocrit level, need for transfusion, Hb level at birth, maximum serum bilirubin, any IVH, and developmental quotient at 2 years of age. Ratings were classified according to the four levels of quality recommended by the GRADE approach: high, moderate, low, and very low quality of evidence.

## Results

### Initial results of the search

**Fig 2:**
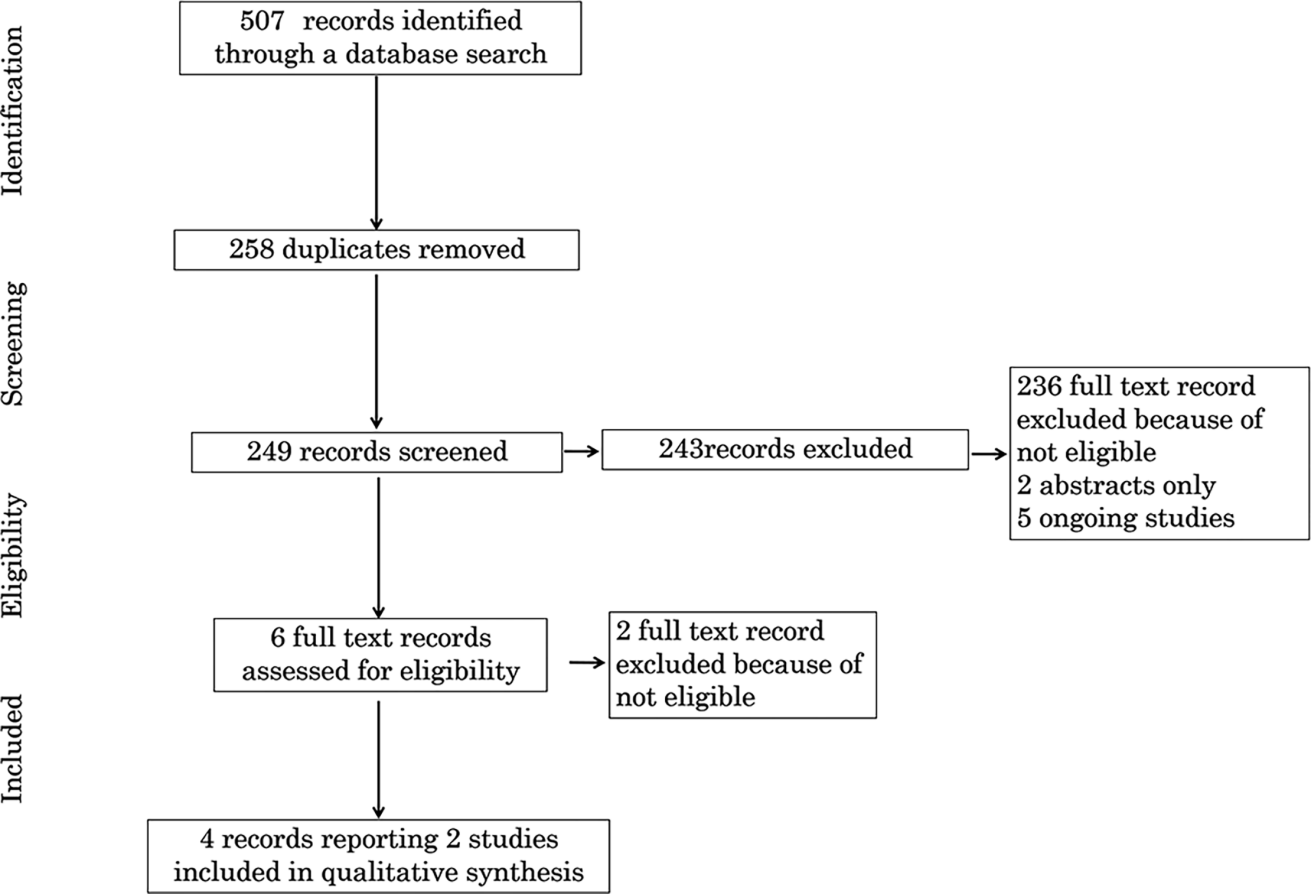
Flow diagram of search results and study selection.

The initial search identified 249 reports, of which 243 were excluded as they did not compare UCM to DCC or were not RCTs. Six reports from four studies met the inclusion criteria, while two studies were excluded. The Argarwal study included term infants and the Kruger study compared DCC with DCC plus UCM. Two of the four included reports were follow-up studies (Fig 2).

### Included studies

Two studies (on 255 preterm infants) were eligible for inclusion in this review, and both studies compared the effects of DCC to UCM [10,11]. However, some differences were observed between the trials regarding participants, settings, and interventions (timing of cord clamping in the experimental and control groups and co-interventions). Differences in both measured outcomes and measurement methods were also noted. The primary outcomes in-hospital mortality, need for transfusion, and initial hematocrit level were not addressed in both studies. In addition, some outcomes were not reported for all participants. Therefore, we include the number of studies and participants for all comparisons.

### Participants

The participants were preterm infants born vaginally or via cesarean delivery. In the study by Katheria (2015), 197 singleton infants born between 23 0/7 and 31 6/7 weeks of gestation were delivered vaginally (n = 43) or by cesarean section (n = 154) [10]. In the study by Rabe et al. (2011), 58 singleton infants born between 24 0/7 and 32 6/7 weeks of gestation were delivered vaginally or by cesarean section [11].

### Settings

Studies were conducted in the United States (Katheria, 2015) or the United Kingdom (Rabe et al., 2011) [10,11].

### Interventions

In the study by Katheria (2015), UCM was performed four times for longer than 2 seconds, with a pause of 1–2 seconds between each milking motion. UCM was compared to DCC, in which the delay was at least 45 seconds. Both methods were conducted by positioning the infant at or about 20 cm below the level of the placenta [10]. In Rabe et al. (2011), UCM was performed four times at a speed of 20 cm for 2 seconds, with 4–5 seconds of cord milking, and was compared to DCC at 30 seconds [11].

### Outcomes

In Katheria (2015), the outcomes were as follows: flow in the superior vena cava within the first 12 hours after the procedure, Hb level at birth (g/dl), polycythemia (hematocrit > 65%), urine output during the first 24 hours after the procedure(ml/kg/hr), need for transfusion, peak bilirubin level (mg/dl), necrotizing enterocolitis, retinopathy requiring surgery, spontaneous perforation of the intestines, corrected oxygen at 36 weeks, any IVH, severe IVH (> grade 3), sepsis (culture-positive), infant mortality, diameter of patent ductus arteriosus (mm), need for pressors, patent ductus arteriosus requiring treatment, and ligation of a patent ductus arteriosus [10]. A follow-up study reported the developmental quotient at 2 years of age (using Bailey III scores) as an outcome [12].

In Rabe et al. (2011), the outcomes were as follows: initial hematocrit level (%), Hb level at 1 hour of age (g/l), blood cord pH, Apgar scores at 5 and 10 minutes, temperature on admission to the neonatal care unit (°C), blood pressure at 4 hours after birth (mmHg), blood sugar on admission to the neonatal care unit (mmol/l), highest serum bilirubin level (mmol/l), time of phototherapy (hours), hematocrit and Hb at 24 hours, day 3, day 7, and weekly thereafter, number of blood transfusions in the first 42 days after delivery, IVH (according to the Papile staging system), number of septic episodes in the first 42 days after delivery, death of the newborn or mother, number of days that the infant required ventilation, number of surfactant treatments, number of days that the infant required oxygen, bronchopulmonary dysplasia (defined as a need for oxygen at 36 weeks of corrected age), retinopathy due to prematurity, necrotizing enterocolitis (according to the Bell staging system), and length of hospital stay [11]. A follow-up study reported the developmental quotient at 2 and 3.5 years of age (using Bailey III scores) as an outcome [13].

### Risk of bias in the included studies

**Fig 3:**
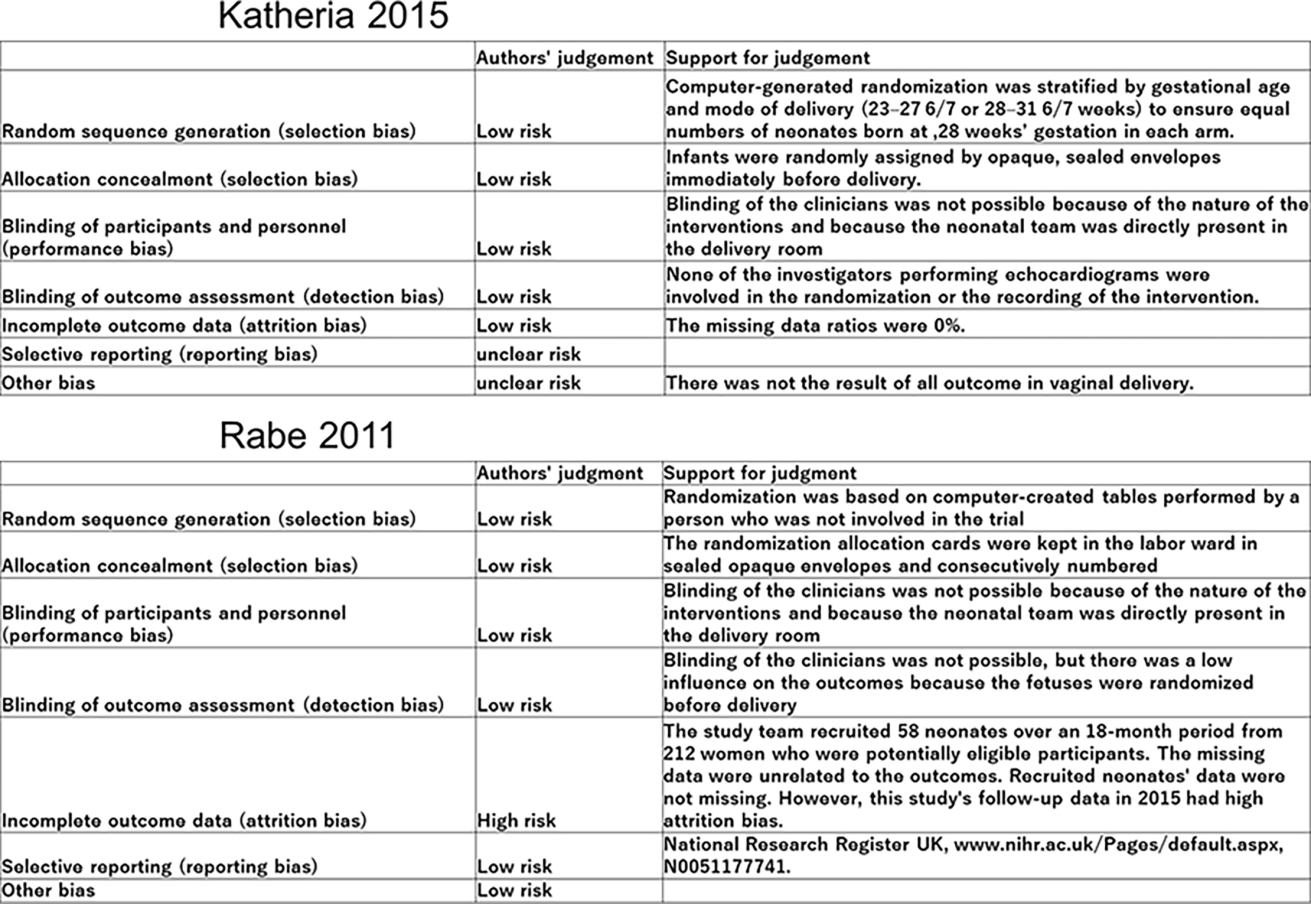
Risk of bias in the included studies.

Risk of bias was identified in all two included studies (Fig 3).

### Allocation

Both trials used computer randomization for allocation and both used sequentially numbered, sealed, opaque envelopes to conceal group allocation, implying low risk of bias.

### Blinding

It was not possible to blind clinicians in either trial due to the nature of the interventions as well as the presence of other clinicians in the delivery room, as per usual practice. However, we judged that lack of blinding was not likely to have any effect (low risk of bias) on the following outcomes: in-hospital mortality, initial hematocrit level, Hb level at birth, maximum serum bilirubin, any IVH, developmental quotient at 2 and 3.5 years of age, polycythemia, mean blood pressure at 4 hours, urine output in the first 24 hours, necrotizing enterocolitis, sepsis, spontaneous intestinal perforation, and Apgar score at 5 min. Alternatively, lack of blinding may have affected the following outcomes: need for transfusion, oxygen dependency at 36 weeks, duration of phototherapy, length of hospital stay, and need for pressors.

### Incomplete outcome data

There were no missing data in Katheria (2015), so the risk of attrition bias was low [10]. In Rabe et al. (2011), 212 potentially eligible infants were recruited over an 18-month period and 58 infants ultimately included in the analysis. There was no attrition for the hospital outcomes (low risk of bias) [11]. However, this study’s follow-up data in 2015 had high attrition bias, as only 39 infants (65%) (UCM: 22 infants, DCC: 17 infants) were included [13].

### Quality of the evidence (GRADE)

The quality of evidence for mortality in the neonatal intensive care unit was rated as low and downgraded due to small sample size. The initial hematocrit level, need for transfusion, Hb level at birth, maximum serum bilirubin, and any IVH were rated as low quality of evidence due to relatively wide 95% CIs and suboptimal sample size. The developmental quotient at 2 years of age was also deemed low quality of evidence because one study had a high risk of bias. Quality of evidence in our review is presented in the GRADE summary of findings table (Table 1).

**Table 1:** Summary of findings.

Umbilical cord milking versus delayed cord clamping of preterm infants						
Population: Preterm infants Setting: USA, UK Intervention: umbilical cord milking Comparison: delay clamping						
Outcomes	Anticipated absolute effects^*^ (95% CI)		Relative effect (95% CI)	№ of participants (studies)	Quality of the evidence (GRADE)	Comments
	**Risk with delay clamping**	**Risk with Milking**				
NICU mortality (ie, mortality until discharge)	91 per 1,000	**40 per 1,000** (13 to 122)	**RR 0.44** (0.14 to 1.34)	212 (2 RCTs)	⨁⨁◯◯ LOW ^a^^,^^b^^‘^	
Initial hematocrit	The mean initial hematocrit in the intervention group was 1.0 higher (2.9 lower to 4.9 higher)		-	58 (1 RCT)	⨁⨁◯◯ LOW ^a^^,^^b^^,^^c^	
Need for transfusion	509 per 1,000	**473 per 1,000** (356 to 621)	**RR 0.93** (0.70 to 1.22)	212 (2 RCTs)	⨁⨁◯◯ LOW ^a^^,^^b^	
Any IVH	146 per 1,000	**66 per 1,000** (29 to 143)	**RR 0.45** (0.20 to 0.98)	255 (2 RCTs)	⨁⨁◯◯ LOW ^a^^,^^b^	
Hb level at birth	The mean birth Hb in the intervention group was 0.43 higher (0.1 lower to 0.96 higher)		-	255 (2 RCTs)	⨁⨁◯◯ LOW ^a^^,^^b^	
Maximum serum bilirubin	The mean maximum serum bilirubin in the intervention group was 0.53 higher (0.17 lower to 1.22 higher)		-	212 (2 RCTs)	⨁⨁◯◯ LOW ^a^^,^^b^	
Developmental quotient of 2 y Bayley scores (≧85) 2 year (Cognitive)	841 per 1,000	**959 per 1,000** (867 to 1,000)	**RR 1.14** (1.03 to 1.26)	174 (2 RCTs)	⨁⨁◯◯ LOW ^a^^,^^d^	

*The risk in the intervention group (and its 95% confidence interval) is based on the assumed risk in the comparison group and the **relative effect** of the intervention (and its 95% CI).

**CI:** Confidence interval; **RR:** Risk ratio; **MD:** Mean difference

GRADE Working Group graades of evidence

**High quality:** We are very confident that the true effect lies close to that of the estimate of the effect

**Moderate quality:** We are moderately confident in the effect estimate: The true effect is likely to be close to the estimate of the effect, but there is a possibility that it is substantially different

**Low quality:** Our confidence in the effect estimate is limited: The true effect may be substantially different from the estimate of the effect

**Very low quality:** We have very little confidence in the effect estimate: The true effect is likely to be substantially different from the estimate of effect

Explanations

a. Optimal sample size not met (imprecision -1).

b. Wide 95%CI (imprecision -1).

c. One study with small sample size (imprecision -1).

d. Risk of bias is high risk (risk of bias -1).

### Effects of the interventions

1. Mortality in the neonatal intensive care unit (i.e., mortality before discharge)

**Fig 4:**
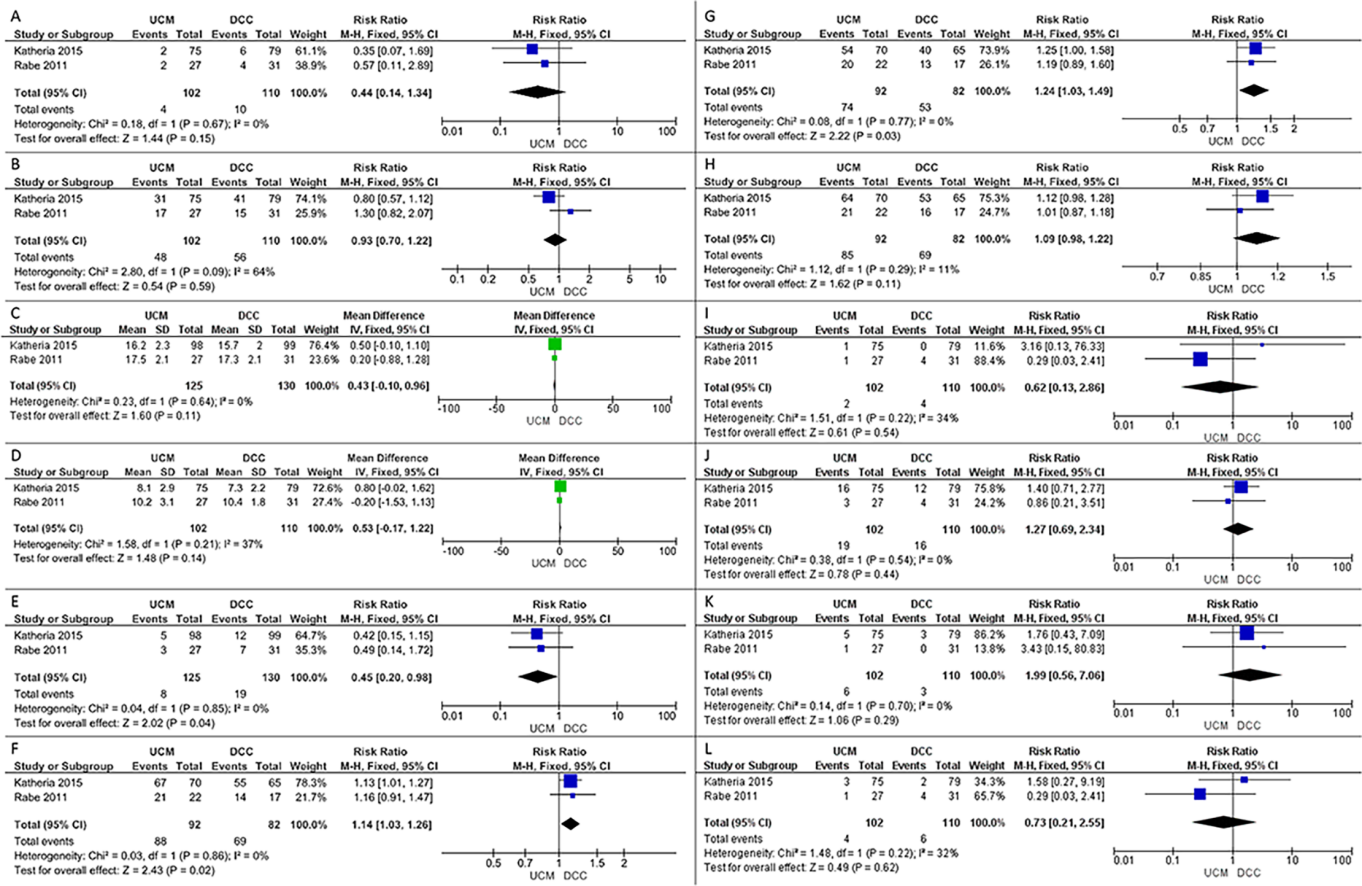
Comparison of umbilical cord milking and delayed cord clamping. (A) Outcome 1: neonatal intensive care unit mortality. (B) Outcome 3: Need for transfusion. (C) Outcome 4: Hb level at birth. (D) Outcome 5: Maximum serum bilirubin. (E) Outcome 6: IVH. (F) Outcome 7: Development quotient using Bailey Ⅲ Cognitive scores at 2 years of age ≧85. (G) Outcome 7: Development quotient using Bailey Ⅲ Language scores at 2 years of age ≧85. (H) Outcome 7: Development quotient using Bailey Ⅲ Motor scores at 2 years of age ≧85. (I) Outcome 12: Necrotizing enterocolitis. (J) Outcome 13: Oxygen dependency at 36 weeks. (K) Outcome 14: Sepsis. (L) Outcome 16: Spontaneous intestinal perforation. UCM: umbilical cord milking; DCC: delayed cord clamping; SD: standard deviation; CI: confidence interval.

In the two studies included in our review, a total of 14 infants out of 212 died in the neonatal intensive care unit before discharge [10,11]. UCM was not associated with a lower number of deaths than DCC (RR 0.44, 95% CI 0.14 to 1.34, low quality of evidence). There was no statistical heterogeneity (I^2^ = 0%) (Fig 4A) [10,11].

2. Initial hematocrit level

The initial hematocrit level was reported for 58 infants in one of the included studies (Rabe et al., 2011). No significant difference was observed between UCM and DCC (MD 1.00, 95% CI -2.90 to 4.90) [11].

3. Need for transfusion

Both included studies reported the need for transfusion (pooled n = 212 infants). No significant difference was observed between UCM and DCC (RR 0.93, 95% CI 0.70 to 1.22, low quality of evidence). There was moderate heterogeneity (I^2^ = 64%) (Fig 4B) [10,11].

4. Hb level at birth

Both studies reported Hb level at birth (pooled n = 255 infants). The mean Hb level at birth did not differ significantly between UCM and DCC (MD 0.43, 95% CI -0.10 to 0.96, low quality of evidence). There was no statistical heterogeneity (I^2^ = 0%) (Fig 4C) [10,11].

5. Maximum serum bilirubin

Both studies reported the maximum serum bilirubin (pooled n = 212 infants). There was no difference between the groups (MD 0.53, 95% CI -0.17 to 1.22, low quality of evidence). There was moderate heterogeneity (I^2^ = 37%) (Fig 4D) [10,11].

6. IVH

Both studies reported on IVH (pooled n = 255). UCM was associated with a lower number of IVH cases than DCC (RR 0.45, 95% CI 0.20 to 0.98, low quality of evidence) and there was no statistical heterogeneity (I^2^ = 0%) (Fig 4E) [10,11].

7. Development quotient using Bailey III scores at 2 and 3.5 years of age

Both studies reported developmental quotients at 2 years of age (pooled n = 174). There was an increased proportion of infants with a Bayley score ≥85 for cognition and language development in the UCM group (Cognitive: RR 1.14, 95% CI 1.03 to 1.26; Language: RR 1.24, 95% CI 1.03 to 1.49; low quality of evidence) and there was no statistical heterogeneity (I^2^ = 0%). However, there was no difference between the groups in the proportion with Bayley scores ≥85 for motor development (RR 1.09, 95% CI 0.98 to 1.22) (Fig 4F, 4G and 4H) [12,13]. One of the included studies (Rabe et al., 2011) also reported developmental quotients at 3.5 years of age for 29 infants. No significant difference was observed between UCM and DCC (MD 0.98, 95% CI 0.76 to 1.25) [13].

8. Polycythemia

The study by Katheria (2015) reported results for polycythemia in 154 infants.

No significant difference was observed between UCM and DCC (RR 0.53, 95% CI 0.10 to 2.79) [10].

9. Mean blood pressure at 4 hours

The study by Rabe et al. (2011) reported the mean blood pressure of 58 infants at 4 hours post-intervention. No significant difference was observed between UCM and DCC (MD 4.00, 95% CI 0.32 to 7.68) [11].

10. Urine output over the first 24 hours after intervention (ml/kg/hr)

The study by Katheria (2015) reported the urine output over the first 24 hours for 154 infants. The urine output was increased in the UCM group compared to DCC (MD 0.43, 95% CI 0.03 to 0.83) [10].

11. Duration of phototherapy (hours)

The study by Rabe et al. (2011) reported the duration of phototherapy for 58 infants. No significant difference was observed between UCM and DCC (MD -5.00, 95% CI -11.45 to 1.45). [11]

12. Necrotizing enterocolitis

Both studies reported the incidence of necrotizing enterocolitis (pooled n = 212 infants). No significant difference in incidence was observed between UCM and DCC (RR 0.62, 95% CI 0.13 to 2.86). There was moderate heterogeneity (I^2^ = 34%) (Fig 4I) [10,11].

13. Oxygen dependence at 36 weeks

Both studies reported on oxygen dependence at 36 weeks (pooled n = 212 infants). No significant difference was observed between UCM and DCC (RR 1.27, 95% CI 0.69 to 2.34). There was no statistical heterogeneity (I^2^ = 0%) (Fig 4J) [10,11].

14. Sepsis

Both studies reported on sepsis (pooled n = 212 infants) and no significant difference was observed between UCM and DCC (RR 1.99, 95% CI 0.56 to 7.06). There was no statistical heterogeneity (I^2^ = 0%) (Fig 4K) [10,11].

15. Length of hospital stay

The study by Rabe et al. (2011) reported the length of hospital stay for 58 infants. No significant difference was observed between UCM and DCC (MD -4.00, 95% CI -17.93 to 9.93) [11].

16. Spontaneous intestinal perforation

Both studies reported on spontaneous intestinal perforation (pooled n = 212 infants). No significant difference was observed between UCM and DCC (RR 0.73, 95% CI 0.21 to 2.55). There was moderate heterogeneity (I^2^ = 32%) (Fig 4L) [10,11].

17. Need for pressors

The study by Katheria (2015) reported on the need for pressors in 154 infants. No significant difference was observed between UCM and DCC (RR 0.53, 95% CI 0.25 to 1.10) [10].

18. Apgar score at 5 min

The study by Rabe et al. (2011) reported Apgar scores at 5 min for 58 infants. No significant difference was observed between UCM and DCC (MD -1.00, 95% CI -2.24 to 0.24) [11].

## Discussion

The current review included data from two trials comparing UCM to DCC on multiple neonatal outcomes in more than 250 preterm infants born between 22 and 32 6/7 weeks of gestation by vaginal or cesarean delivery. Our systematic review and meta-analysis shows that UCM has no potential harms compared to DCC and suggests that UCM is associated with a lower incidence of IVH compared to DCC. However, there was no difference between these two interventions for any of the primary outcomes (mortality in the neonatal intensive care unit, initial hematocrit level and need for transfusion: low quality evidence). This reduced IVH risk may stem from the increased circulating volume conferred by UCM. Further studies are necessary to confirm the short- and long-term effects of UCM versus DCC on infants born between 33 and 36 6/7 weeks of gestation. One study DCC plus UCM demonstrated a mortality advantage over only DCC despite both groups experiencing the same delay in resuscitation (30 seconds), although the difference was not statistically significant [14]. The volume of placental-fetal transfusion may be related to the decrease in mortality. The placenta can hold up to 40% of the total circulating fetal blood volume because the cord vein alone has a blood volume of 15 to 20 mL [15]. A recent randomized study of DCC (delay of 30 to 120 seconds) in 46 preterm neonates at less than 33 weeks of gestation [16] showed an 18% blood volume increase using DCC compared to ICC (mean 74.4 mL/kg, range 45–103 mL/kg vs. 62.7 mL/kg, range 47–77 mL/kg; *P* < 0.001). However, another RCTs [17] reported that DCC had no effect on systemic blood flow measured as mean lowest superior vena cava flow over the first 24 hours in preterm infants born at less than 30 weeks’ gestation compared to ICC. Therefore, UCM provides extra volume earlier than DCC and a lot earlier than standard blood transfusion, which may have beneficial physiological effects that ultimately improve outcome. Larger-scale studies are needed to confirm these findings. It is possible that DCC plus UCM increases the amount of placento-fetal transfusion compared to DCC alone for preterm infants. However, Krueger did not find a difference in hematocrit between UCM plus DCC and UCM, so again larger high-quality trials with sufficient power to assess significant differences in placenta-fetal transfusion volume are required.

A previous meta-analysis of six studies (587 infants) comparing UCM and ICC for blood pressure stabilization in preterm infants found that UCM was associated with lower blood transfusion exposure (RR 0.74, 95% CI 0.61 to 0.90), IVH incidence (RR 0.71, 95% CI 0.55 to 0.91), necrotizing enterocolitis (RR 0.60, 95% CI 0.39 to 0.93), and infant mortality (RR 0.50, 95% CI 0.30 to 0.86) [18]. Another systematic review and meta-analysis comparing UCM or DCC to ICC suggested that enhanced placental transfusion at birth in very preterm neonates reduces overall mortality, lowers IVH risk, decreases the need for blood transfusion, and improves neonatal outcomes compared to early cord clamping [19]. A low Hb level at birth was shown to be significantly associated with mortality and the need for blood transfusion in preterm infants born at ≤ 32 weeks of gestation [20]. Our analysis found no significant difference in Hb level at birth between UCM and DCC. For the other two related primary outcomes, need for transfusion and the initial hematocrit level, the CIs for the RR were relatively wide; therefore, there is currently insufficient evidence that UCM holds any advantage over DCC. Polglase et al. (2015) reported that the establishment of ventilation prior to umbilical cord clamping facilitated a smooth transition to systemic and cerebral oxygenation following birth in preterm lambs [21]. Thus, resuscitation onset prior to umbilical cord clamping may improve systemic and cerebral oxygenation in preterm infants. Duley et al. (2016) reported that clamping after at least 2 min and immediate neonatal care with cord intact at very preterm birth may improve outcome. Neonatal stabilisation and resuscitation can be provided with the cord intact. This result suggests that DCC seems to be able to fit preterm infants require resuscitation immediately after birth [22]. But this study compared with DCC and ICC, and DCC group was included cord clamping within 20 sec. The effect of resuscitating preterm infants with an intact cord needs to be addressed in a large multicentrer trial.

Both studies in this meta-analysis reported on neurodevelopmental outcomes of children at 2 years of age. UCM resulted in an increased proportion of infants with normal Cognitive and Language Bayley scores at 2 years of age compared to DCC despite no clear difference in Apgar scores or umbilical artery blood gas parameters on admission to the neonatal care unit (although these outcomes were not reported for both trials). In a recent comparison of DCC to UCM in term-born Indian infants [23], DCC at 60−90 seconds resulted in no significant differences in ferritin level, hemoglobin levels, and growth parameters at 12 months of age compared to UCM. It is possible that the lower incidence of IVH may account for the improved developmental quotients observed in our study.

In the pooled results, most outcomes had relatively large CIs, so the results need to be interpreted carefully. Nonetheless, these results warrant larger multicenter trials to compare approaches for successful placental transfusion, including DCC and UCM.

## Conclusions

### Implications for practice

This study demonstrated low quality evidence for reduced IVH and improved neurodevelopmental outcomes with UCM. However,there was no difference for any of the primary outcomes and hence there is insufficient evidence to state whether one technique results in better outcomes than the other. Ensuring healthy growth and development among preterm children is important, and long-term follow-up of child participants in clinical trials is needed to create reliable evidence for clinical recommendations.

### Implications for research

Our review describes the benefits of UCM for achieving rapid placental transfusion. However, studies conducted so far in this area are small scale, have some risk of bias, do not report data on all primary outcomes, and have unclear long-term outcomes. Larger high-quality trials with sufficient power to assess important differences in primary outcomes and long-term follow-up are required so that approaches can be accurately compared and assessed. Larger collaborative studies on an international scale are needed to develop a universal approach to improving the delivery and resuscitation of preterm infants. More data on maternal outcomes, respiratory outcomes, and long-term neurodevelopment outcomes are also needed.

## Supporting information

S1 Table Search strategies. We used this search strategies.
(DOC)

S2 Table PRISMA 2009 checklist. (DOC)
