# Supplementary material for: Benefits of umbilical cord milking versus delayed cord clamping on neonatal outcomes in preterm infants: A systematic review and meta-analysis
Source: PLoS One. 2018 Aug 30;13(8):e0201528. doi: 10.1371/journal.pone.0201528 (PMC6116944; doi:10.1371/journal.pone.0201528)
Supplement: S1 Table — We used this search strategies. (DOC) [file pone.0201528.s001.doc]

**1: Search Strategies**

***A. CINAHL***

S8 S5 AND S6 AND S7 Limiters - Exclude MEDLINE records

S7 ( (MH "Randomized Controlled Trials") OR (MH "Crossover Design") OR (MH "Random Assignment") OR (MH "Triple-Blind Studies") OR (MH "Double-Blind Studies") OR (MH "Single-Blind Studies") ) OR TI ( Random* OR Crossover OR "Cross Over" OR ((Double OR Single OR Triple) N1 Blind*) OR (Control* N2 Trial*) ) OR AB ( Random* OR Crossover OR "Cross Over" OR ((Double OR Single OR Triple) N1 Blind*) OR (Control* N2 Trial*) )

S6 ( (MH "Infant, Premature") OR (MH "Childbirth, Premature") OR (MH "Labor, Premature") OR (MH "Infant, Premature, Diseases+") ) OR TI ( Prematur* OR Preterm OR Immatur* ) OR AB ( Prematur* OR Preterm OR Immatur* )

S5 S3 OR S4

S4 (MH "Umbilical Cord Clamping")

S3 S1 AND S2

S2 TI ( Clamp* OR Milk* ) OR AB ( Clamp* OR Milk* )

S1 ( (MH "Umbilical Cord+") OR (MH "Umbilical Cord Care") ) OR TI ( Umbilical OR Cord OR Cords ) OR AB ( Umbilical OR Cord OR Cords )

***B. Cochrane Library***

([mh "Umbilical Cord"] OR Umbilical OR Cord OR Cords) AND (Clamp* OR Milk*) AND ([mh "Obstetric Labor, Premature"] OR [mh "Mortality, Premature"] OR [mh "Infant, Premature, Diseases"] OR [mh "Infant, Premature"] OR Prematur* OR Preterm OR Immatur*) in Trials

***C. EMBASE***

1. Exp Umbilical Cord/ OR (Umbilical OR Cord OR Cords).af.
2. (Clamp$ OR Milk$).af.
3. Exp Prematurity/ OR Exp "Immature and Premature Labor"/ OR Premature Mortality/ OR (Prematur$ OR Preterm OR Immatur$).af.
4. Crossover-Procedure/ OR Double-Blind Procedure/ OR Randomized Controlled Trial/ OR Single-Blind Procedure/ OR (Random$ OR Factorial$ OR Crossover$ OR (Cross Over$) OR Cross-Over$ OR Placebo$ OR (Doubl$ adj Blind$) OR (Singl$ adj Blind$) OR Assign$ OR Allocat$ OR Volunteer$).mp.
5. 1 AND 2 AND 3 AND 4
6. Exp Animals/ OR Exp Invertebrate/ OR Animal Experiment/ OR Animal Model/ OR Animal Tissue/ OR Animal Cell/ OR Nonhuman/
7. Human/ OR Normal Human/ OR Human Cell/
8. 6 NOT 7
9. 5 NOT 8
10. Limit 9 to MEDLINE

***D. MEDLINE***

1. Exp Umbilical Cord/ OR (Umbilical OR Cord OR Cords).af.
2. (Clamp$ OR Milk$).af.
3. Exp "Obstetric Labor, Premature"/ OR Exp "Mortality, Premature"/ OR Exp "Infant, Premature, Diseases"/ OR Exp "Infant, Premature"/ OR (Prematur$ OR Preterm OR Immatur$).af.
4. (Randomized Controlled Trial OR Controlled Clinical Trial OR Pragmatic Clinical Trial).pt. OR (Randomi?ed OR Randomly OR Placebo OR Trial OR Groups).ab. OR Drug Therapy.fs. NOT (Animals NOT (Humans and Animals)).sh.
5. 1 AND 2 AND 3 AND 4

***E. PubMed***

("Umbilical Cord"[Mesh] OR Umbilical[All Fields] OR Cord[All Fields] OR Cords[All Fields]) AND (Clamp*[All Fields] OR Milk*[All Fields]) AND ("Obstetric Labor, Premature"[Mesh] OR "Mortality, Premature"[Mesh] OR "Infant, Premature, Diseases"[Mesh] OR "Infant, Premature"[Mesh] OR Prematur*[All Fields] OR Preterm[All Fields] OR Immatur*[All Fields]) AND (Randomized Controlled Trial[pt] OR Controlled Clinical Trial[pt] OR Pragmatic Clinical Trial[pt] OR Randomized[tiab] OR Randomised[tiab] OR Placebo[tiab] OR Randomly[tiab] OR Trial[tiab] OR Groups[tiab]) NOT MEDLINE[sb]
